# Clinical evaluation of the Sorin Xtra® autotransfusion system

**DOI:** 10.1177/0267659112442222

**Published:** 2012-07

**Authors:** EP Overdevest, PWJ Lanen, JCM Feron, JWH van Hees, MESH Tan

**Affiliations:** 1 Department of Extracorporeal Circulation and Blood Management, Catharina Hospital Eindhoven, Eindhoven, the Netherlands; 2 Department of Cardiothoracic Surgery, Catharina Hospital Eindhoven, Eindhoven, the Netherlands

**Keywords:** autotransfusion, cell saver, red blood cells

## Abstract

The performance of the Sorin Xtra® Autotransfusion System (ATS) was studied in 62 patients undergoing coronary artery bypass grafting. Blood was collected intraoperatively and washed using three different wash sets in 4 groups. Both collected and washed blood were analysed for hemoglobin levels and hematocrit, concentrations of proteins, albumin, heparin and plasma free hemoglobin (PFH) were determined, erythrocytes, platelets and leukocytes were counted.

Hematocrit measurements of the Xtra® were compared with laboratory measurements to study the accuracy of the Xtra® hematocrit sensor. In addition, the red blood cell recovery rate and elimination rates were calculated to evaluate the clinical performance of the Xtra®.

The Xtra® ATS produced a volume of concentrated red blood cells with an average hematocrit from 58% to 63%, depending on the size of the bowl and the chosen default program. In all bowl sizes and programs, the Xtra® Hct-out measurement underestimated the CELL-DYN measurement by approximately 15%.

The calculated recovery rates for red blood cells (RBC) in the 4 groups ranged from 86.7% to 91.6%. Elimination rates were calculated in each group for proteins (96.8-99.2%), albumin (96.4-98.7%), plasma free hemoglobin (83.6–91.2%), heparin (98.8-99.9%), platelets (82.4-94.3%) and white blood cells (28.6-42.3%).

The Xtra® ATS can be appealing for its performance by producing high hematocrit levels in the washed RBC volume, while keeping RBC recovery rate at the same high level (≈ 90%) as in its predecessor, the Electa® Autotransfusion System.

## Introduction

The first commercially available red blood cell-salvaging device was manufactured by the Haemonetics Corporation in 1974^1^. The latest introduction of a new autotransfusion system occurred in 2010, when the Sorin Group launched the Sorin Xtra®, a new generation autotransfusion device, equipped with a graphic-colored touch-screen user interface, an improved data management system (DMS) and a powerful vacuum pump. The new system is using a modified disposable bowl set for easier and faster setup and improved performances. This study examines the clinical performance of the new device.

## Materials and Methods

Between June 15^th^ and October 15^th^ 2010, 62 patients underwent coronary artery bypass grafting using the Xtra® autotransfusion device for pre- and postoperative collection of blood loss in the OR, intra-operative collection of heparinized suction blood and processing of residual blood volume of the heart-lung machine (HLM).

Blood was collected throughout the whole cardiac procedure. At the end of the surgical procedure, after the residual volume was transferred into the collection reservoir and a sufficient amount of blood volume was available in the reservoir to ensure complete filling of the selected bowl for at least one cycle, processing of the collected volume was initiated.

### Wash sets and default programs

In this study, the 225ml, the 175ml and the 125ml wash sets were used to process the collected volume. For processing, a manufacturer’s in-built default program can be chosen. The optimal default program (Popt) was used for the each wash set. This program uses two different pump speeds during the filling phase. For a fourth group, using a 225ml wash set, the standard default program was adjusted (Pstd_P(lus)). Compared to the traditional standard default program, the wash volume and wash speed were reduced. The different programs, volumes and flow settings are shown in Table 1.

**Table 1.** Default Programs

Wash set (ml)	Program	Q*-prime (ml/min)	Q-wash (ml/min)	V**-wash (ml)	Q-empty (ml/min)
225	Popt	400/250	500	1000	400
225	Pstd_P***	350	450	600	450
175	Popt	550/300	450	1000	400
125	Popt	450/250	250	800	300

* Q = speed ** V = volume *** Pstd_P = Pstd_Plus

### Sampling

During the set up of the disposable collection reservoir and wash set, sampling ports were built in for taking samples from the reservoir and the re-infusion bag, without the use of vacuum.

Prior to blood processing, the collection reservoir was carefully shaken to provide a homogenous sample, representing average values in the reservoir. A volume between 100 and 200ml was pumped into the bowl before taking a sample. Using a 20ml syringe, 18ml of blood was slowly aspirated at the sample port close to the collection reservoir. An 18ml blood sample was used to fill 4 Vacutainer™ tubes (Becton Dickinson, Oxford, Oxon, UK). However, a vacuum was used only in the citrate tube (blue cap) to obtain an exact ratio between blood and citrate (4.5ml and 0.5ml, respectively) for anti-Xa detection to determine heparin levels. Two heparin tubes (green cap) were cautiously filled with 4.0ml of blood each, after removal of their caps to prevent induction of hemolysis. One tube was used to determine hemolysis by measuring free hemoglobin. The other heparin tube was used to determine albumin and total protein levels. A fourth EDTA-tube (purple cap) was filled with 4ml of blood for hemoglobin (Hb), hematocrit (Hct), red blood cell (RBC), platelet and leukocyte counts, without the use of vacuum.

The second sample in each procedure was taken from the re-infusion bag. After running the first full cycle, red blood cells were collected in the re-infusion bag. Also, the red blood cell line to the re-infusion bag was emptied in order to collect the complete volume of RBCs of the first cycle in the re-infusion bag. After gently mixing the RBC volume, an 18ml sample was slowly aspirated from the sample port in a 20ml syringe. Similarly, as for the first sample, 4 tubes were filled, using the vacuum of the Vacutainer™ tube for the citrate tube only, to determine anti-Xa. Immediately after filling, all samples were transferred to the laboratory to ensure immediate processing.

### Laboratory instruments and hematocrit detector

For all cell counts (red blood cells, platelets and white blood cells), the CELL-DYN Sapphire® (Abbott Diagnostics, Montreal, Québec, Canada) laboratory instrument was used. Plasma protein levels (total proteins, albumin, PFH) were measured using the Advia® 1650 (Siemens Healthcare Diagnostics, Deerfield, IL, USA). Heparin activity was assessed using a dedicated anti-Xa assay (Roche Stago) on a STA-R Evolution® (Roche Diagnostics, Basel, Switzerland). In short, patient samples are incubated with excess coagulation factor Xa and antithrombin. The heparin present in the sample (or wash solution) binds the factor Xa that is no longer capable of cleaving a chromogenic substrate. The unbound factor Xa is still capable of cleaving the substrate that will release para-nitroallanine (pNA). The amount of pNA formed and measured spectrophotometrically is inversely linear to the amount of heparin (or anti-Xa activity) present in the sample, irrespective of the amount of (active) Xa or antithrombin present in the sample, due to the excess added.

The hematocrit values measured by the Xtra® are calculated using an optical method in which a light signal is emitted by a light-emitting diode (LED). Light is absorbed by hemoglobin and oxyhemoglobin, and residual light is detected by a sensor opposite the light source. The hematocrit of the Xtra® is calculated, based on the amount of light detected by the sensor after calibration with laboratory values.

### Blood volume management

During cardiopulmonary bypass, a closed circuit was used. All activated pericardial and pleural suction blood was collected in the Xtra® collection reservoir. The final residual volume of the heart-lung machine consisted, in most cases, between 500 and 1000ml of autologous blood, after pumping residual volume as much as possible into the patient prior to arterial decannulation to prevent unnecessary loss of coagulation proteins as a result of washing large blood volumes. A volume between 300 and 500ml was handed over to the anesthesia nurse as infusion volume during the administration of protamine. The remaining residual volume was pumped into the collection reservoir by flushing the heart-lung machine circuit with at least 1.5 liters of saline.

During each case, all procedure parameters were registered on clinical research forms and a complete procedure report was printed. Patient procedure data remained stored in the Xtra® autotransfusion device, where they can be accessed at any time during or after the operation.

## Results

### Xtra data

In Table 2, the data from 62 patients are shown. In Table 3, the volumes of processed and recovered red blood cells are shown, both of the first cycle (1^st^ cycle volume and RBC 1^st^ cycle) and of the whole procedure (total processed and total RBC). Also, the time to run the first cycle and the RBC production speed are shown. In a 225ml wash set, using a modified Pstd_Plus default program, the time to run a full cycle is reduced compared to the Popt program. Within a specific default program, different parameters are responsible for the cycle time, namely, the hematocrit of the blood volume entering the bowl, the fill, wash and empty speed and the wash volume. Except for hematocrit, all other speeds and volumes are preset in the default program by the manufacturer. The time for a full cycle can only be compared when all these parameters are similar for the different groups (Tables 1 and 4). Therefore, the average volume of washed RBCs produced per minute is only indicative of what can be achieved with a certain default program and bowl size. The average hematocrit was similar in all groups.

**Table 2.** Patient Data

Group	Popt225 (n=15)	Pstd_P225 (n=15)	Popt175 (n=17)	Popt125 (n=15)
Sex (M/F)	12/3	10/5	13/4	10/5
Age (years)	69.8 (8.2)	62.9 (8.1)	63.5 (11.8)	64.9 (12.4)
B.S.A. (m^2^)	1.91 (0.3)	1.94 (0.2)	1.96 (0.2)	1.99 (0.2)
B.M.I. (kg/m2)	27.9 (5.4)	25.9 (4.1)	26.7 (3.8)	27.7 (3.5)

( ): standard deviation.

**Table 3.** Xtra data

Group	Popt225 (n=15)	Pstd_P225 (n=15)	Popt175 (n=17)	Popt125 (n=15)
1^st^ cycle volume (ml)	1298 (484)	1081 (295)	816 (298)	592 (173)
RBC 1^st^ cycle (ml)	230 (14)	227 (8)	157 (7)	123 (3)
Total processed (ml)	2526 (437)	1864 (609)	2114 (553)	1846(607)
Total RBC (ml)	434 (124)	388 (108)	415 (165)	379 (137)
1^st^ cycle time (min)	8.3 (1.6)	6.1 (1.1)	6.1 (1.3)	6.7 (0.8)
RBC (ml/min)	27.7	37.2	25.7	18.4

( ): Standard deviation.

**Table 4.** Hematocrit Xtra® versus Cell-Dyn Sapphire (CDS)

Group	Xtra®-in (n=62)	CDS-in (n=62)	Xtra®-out (n=62)	CDS-out (n=62)
Popt225	0.13 (0.03)	0.16 (0.05)	0.53 (0.03)	0.63 (0.03)
Pstd_P225	0.13 (0.02)	0.15 (0.03)	0.53 (0.02)	0.61 (0.03)
Popt175	0.13 (0.03)	0.15 (0.04)	0.52 (0.02)	0.58 (0.01)
Popt125	0.13 (0.02)	0.15 (0.03)	0.54 (0.03)	0.60 (0.04)

( ): Standard deviation.

### Hematocrit

In Table 4, the average hematocrit of the processed volume in the first cycle and the average hematocrit of the volume in the re-infusion bag are shown. The highest hematocrit can be achieved with the 225ml wash set, using the Popt default program. The average hematocrit values measured by the Xtra® and the CELL-DYN Sapphire® are shown in Table 4. For all bowl sizes and programs, the Xtra® Hct-out measurement underestimates the CELL-DYN measurement by approximately 15%.

### Recovery and elimination rates

**Figure 1.**
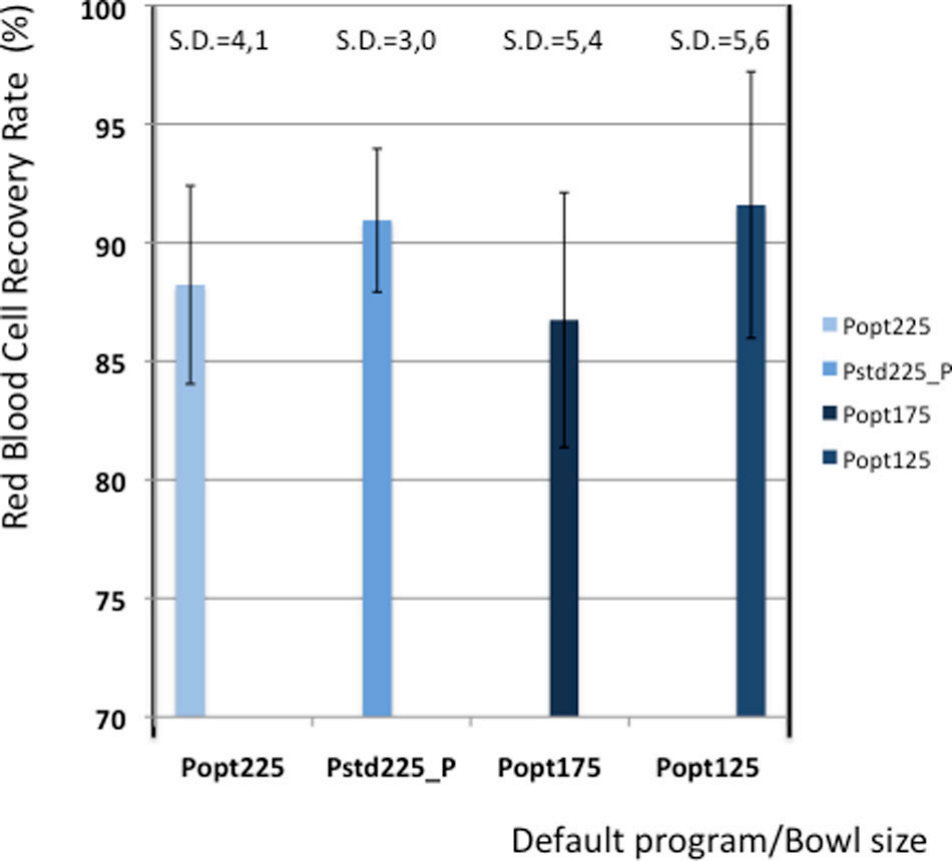
Red blood cell recovery.

Figure 1 shows the average recovery of red blood cells in each group. The highest recovery is achieved in the 125ml bowl, also with the highest standard deviation. The 225ml bowl, using the Pstd_Plus default program shows a high recovery with a smaller standard deviation.

**Figure 2.**
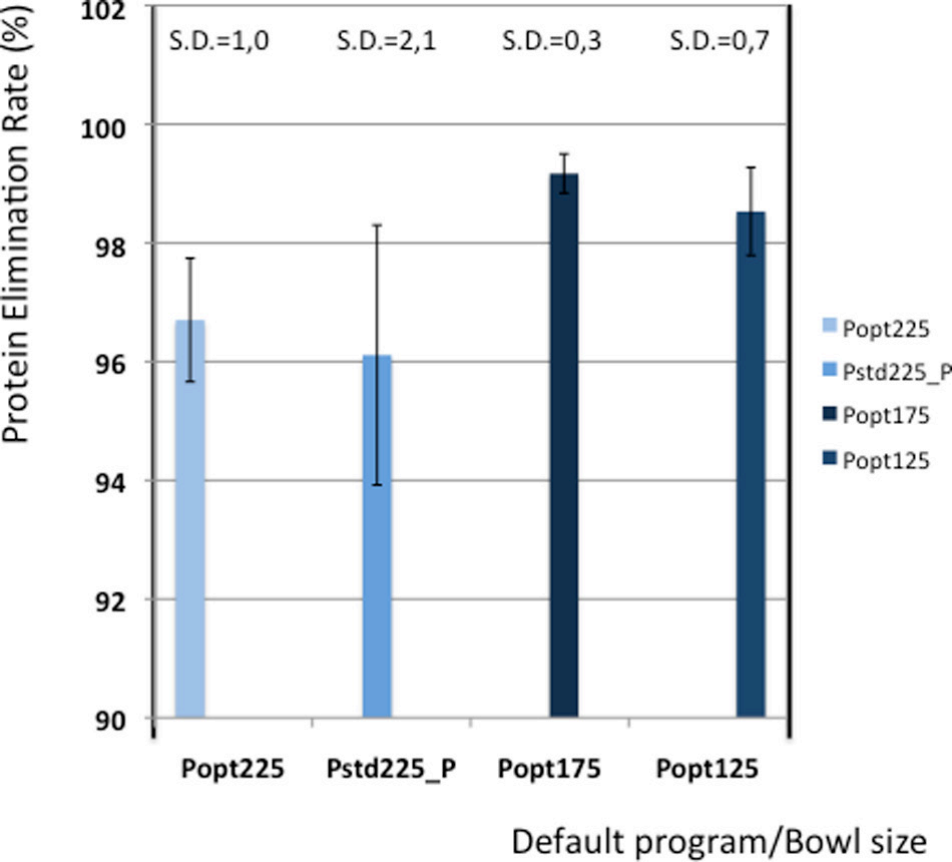
Elimination of proteins.

**Figure 3.**
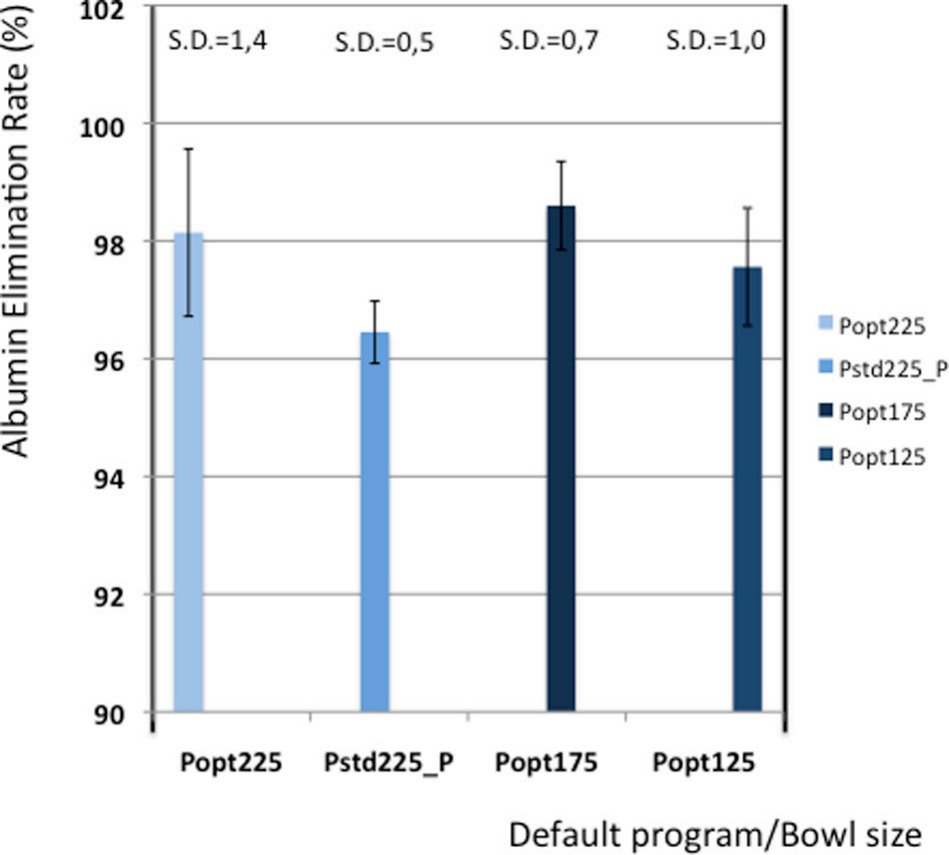
Elimination of albumin.

**Figure 4.**
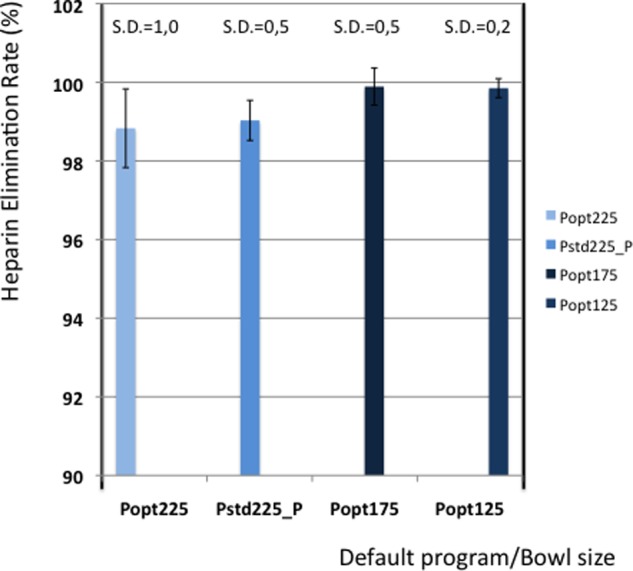
Elimination of heparin.

**Figure 5.**
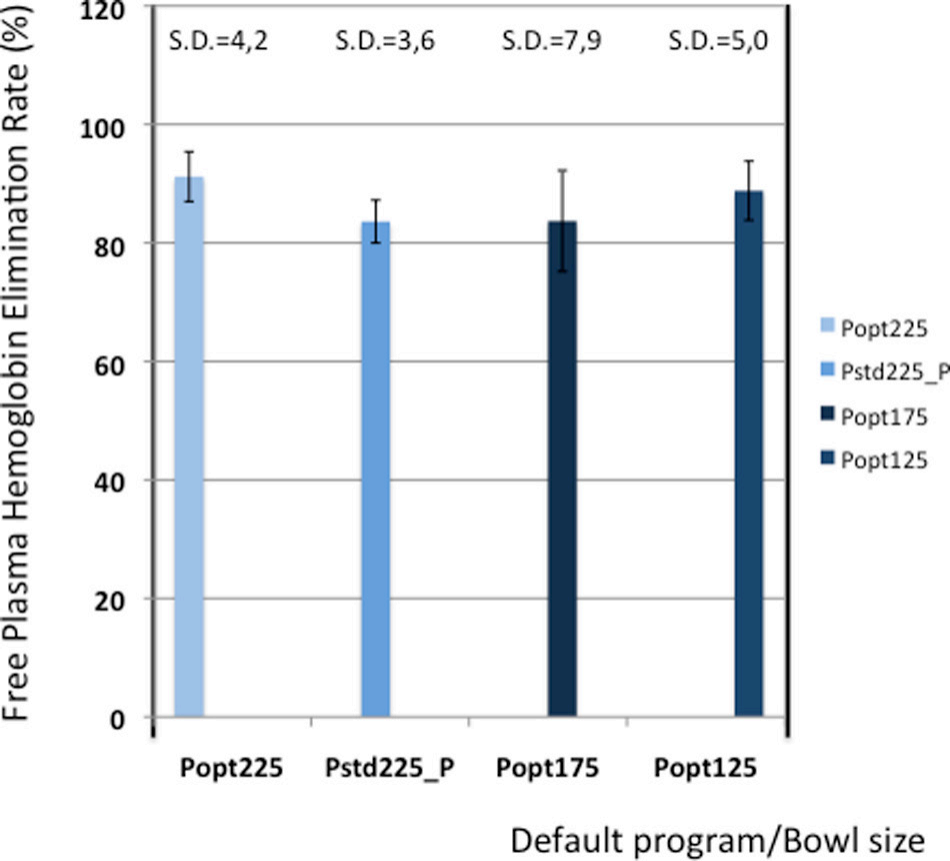
Elimination of plasma free hemoglobin.

**Figure 6.**
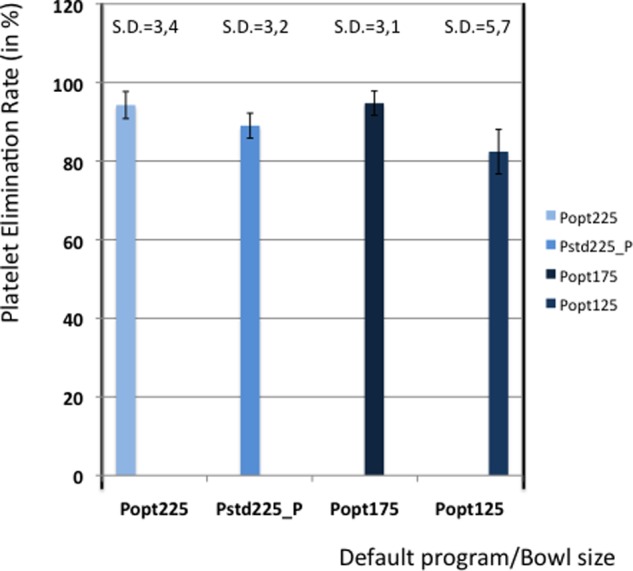
Elimination of platelets.

**Figure 7.**
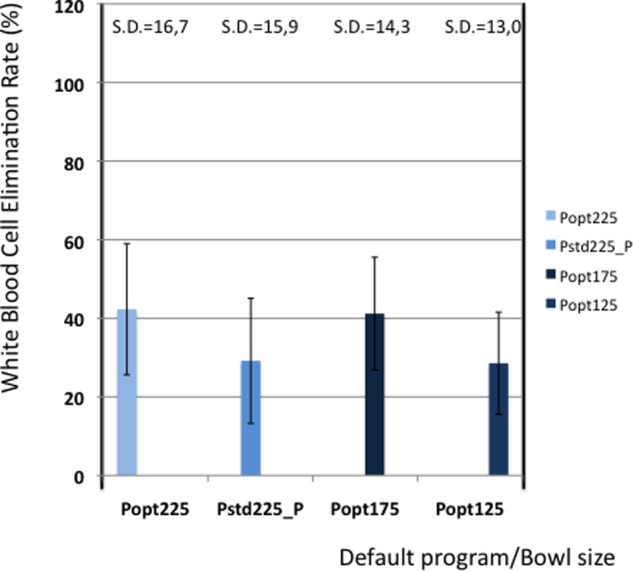
Elimination of white blood cells.

Table 5 shows the elimination values of plasma proteins, albumin, heparin and PFH. In Figures 2 and 3 the elimination rates of total plasma proteins and albumin are shown. The trend in eliminating total proteins is similar to the elimination of albumin. Elimination of heparin, as shown in Figure 4, was between 98 and 100% in all patients. The elimination of PFH was expected to follow the same trend as the elimination of plasma protein removal. However, this was not confirmed in this study (Figure 5). In Figure 6, the elimination rates of platelets are shown. The elimination rates of leukocytes are shown in Figure 7.

**Table 5.** Elimination rates (in %)

Group	E.R.proteins (n=62)	E.R.albumin (n=62)	E.R.heparin (n=62)	E.R.PFH (n=62)
Popt225	98.3 (1.0)	98.1 (1.4)	98.8 (1.0)	91.2 (4.2)
Pstd_P225	96.8 (2.1)	96.5 (0.5)	99.0 (0.5)	84.6 (3.6)
Popt175	99.2 (0.3)	98.6 (0.8)	99.9 (0,5)	83.7 (8.5)
Popt125	98.5 (0.7)	97.6 (1.0)	99.9 (0.2)	88.8 (5.0)

( ): Standard Deviation.

## Discussion

The clinical performance of the Xtra® is excellent with regard to the achieved hematocrit levels in the RBC re-infusion volume. An underestimation of approximately 15% was noticed between the Xtra® hematocrit measurement and the one resulting from the CELL-DYN® on the same samples. Based on these results, the Sorin Group has released a new software version in which the calibration curve of the Xtra® hematocrit sensor has been fine-tuned for higher hematocrit values to improve accuracy. This new software version is presently under evaluation.

The hematocrit values and the red blood cell recovery rate show a high reproducibility in each bowl size due to the presence of a double buffy coat sensor. The improved technology emphasizes the importance of an efficient recovery, especially in the presence of higher hematocrit values. It is due to this technology that the recovery rates of the Xtra® remain in the same range as its predecessor, the Electa.

With regard to recovery and elimination rates, it should be emphasized that these values are calculated using the volumes generated by the Xtra®. Volume calculations by the Xtra® depend on pump rotations and plastic tubing diameter, affected by tolerances that can cause slight deviations. However, in this study we used the same device for all measurements and any error in pump rotation volume will be constant for all measurements.

The elimination of plasma contaminants, proteins and heparin is according to expectations. Compared with other devices in previous studies, the Xtra® performs adequately^3^. With regard to PFH removal, we were not able to generate reliable results. Elimination of PFH is expected to follow the same trend as the removal of plasma proteins and albumin, although this was not shown in this study. Nevertheless, the results on PFH removal are presented in this paper, but they should be interpreted with caution. Although the different steps in PFH measurements were studied with regard to their influence on hemolysis, further attention is required for the validation of PFH measurements with regard to sampling, sample handling, sample turn-around-time, centrifugation time and centrifugation speed.

Similarly, the platelet and leukocyte elimination rates should be interpreted with caution. Platelet counts were performed on the CELL-DYN® laboratory instrument that measures platelets by cell count and by impedance. The cell count was disturbed by structures that were counted as platelets causing a difference in the two measurements. Determination of real platelet numbers was performed in 3 cases by measuring anti-CD61 marker, a protein that can be expressed on platelets. These measurements showed that the real platelet count could be up to 40% less than the measured values by the cell counter method. The determination of leukocyte numbers seemed more accurate, but also leukocyte counts showed a difference between cell count and impedance measurement.

When comparing the results of the different programs, it seems that a reduction of the wash volume in the Pstd_Plus default program does not affect the elimination rates. The Pstd_Plus program also looks significantly faster than the other programs. Taking into account the limitations of the parameters “time to run the 1^st^ cycle” and the “RBC volume per minute” speed, the faster cycle and RBC production speed must be the result of a reduced wash volume. During surgery, this means that recovered blood can be processed faster, without affecting the quality of the procedure.

The perceived benefit of the dual fill speed of the P_opt_ default program is an increased hematocrit. However, the effect on hematocrit, while comparing the result of the two programs for the 225ml bowl, seems limited in this study. A potential risk of a high hematocrit in the bowl is a lower red cell recovery, due to loss of red cells in the wash phase. The Popt program might be improved by using the slower wash speed of the Pstd_Plus program, or even slower, to reduce the loss of red cells and further increase its RBC recovery, keeping in mind that differences in RBC recovery between Popt and Pstd_Plus are not significant.

According to the author, an ideal program for processing blood in cardiac surgery might comprise dual fill speeds, a slower wash speed, a reduced wash amount and a faster empty speed than the present Popt program.

Perhaps similar in importance as its performance, the Xtra® is equipped with today’s technology. The user interface and data management system provide export options and can record data that fulfill the present requirements^4^.

In conclusion, the Xtra® Autotransfusion System can appeal due to its performance in producing high hematocrit levels in the RBC volume while keeping RBC recovery rates at an adequate level.
